# Bi-Planar Trajectory Tracking with a Novel 3DOF Cable Driven Lower Limb Rehabilitation Exoskeleton (C-LREX)

**DOI:** 10.3390/s23031677

**Published:** 2023-02-03

**Authors:** Rajan Prasad, Marwan El-Rich, Mohammad I. Awad, Sunil K. Agrawal, Kinda Khalaf

**Affiliations:** 1 Department of Mechanical Engineering, Khalifa University of Science Technology & Research, Abu Dhabi P.O. Box 127788, United Arab Emirates; 2 Healthcare Engineering Innovation Center, Khalifa University of Science Technology & Research, Abu Dhabi P.O. Box 127788, United Arab Emirates; 3 Department of Biomedical Engineering, Khalifa University of Science Technology & Research, Abu Dhabi P.O. Box 127788, United Arab Emirates; 4 Khalifa University Center for Autonomous Robotic Systems (KUCARS), Khalifa University of Science Technology & Research, Abu Dhabi P.O. Box 127788, United Arab Emirates; 5 Department of Mechanical Engineering and Rehabilitation and Regenerative Medicine, Columbia University, New York, NY 10032, USA

**Keywords:** cable driven, exoskeleton, lower limb rehabilitation, hip adduction, bi-planar trajectory, optimized routing

## Abstract

Although Cable-driven rehabilitation devices (CDRDs) have several advantages over traditional link-driven devices, including their light weight, ease of reconfiguration, and remote actuation, the majority of existing lower-limb CDRDs are limited to rehabilitation in the sagittal plane. In this work, we proposed a novel three degrees of freedom (DOF) lower limb model which accommodates hip abduction/adduction motion in the frontal plane, as well as knee and hip flexion/extension in the sagittal plane. The proposed model was employed to investigate the feasibility of using bi-planar cable routing to track a bi-planar reference healthy trajectory. Various possible routings of four cable configurations were selected and studied with the 3DOF model. The optimal locations of the hip cuffs were determined using optimization. When compared with the five-cable routing configuration, the four-cable routing produced higher joint forces, which motivated the future study of other potential cable routing configurations and their ability to track bi-planar motion.

## 1. Introduction

A variety of lower-limb robotic devices have been designed in recent years for stroke rehabilitation, with the majority generating/assisting limb motion by employing direct actuation (placing an actuator near the joint to generate joint motion). This design approach, however, produces additional inertia and inertial vibration on the limbs; assumes the knee joint as a one DOF pin joint; imposes unnecessary stress and moment/reactions on the knee joint [1]; and causes discomfort to the user. Moreover, the addition of extra weight to the already afflicted limb further challenges the rehabilitation of these patients. Some devices, such as LOPES II [2], implement the concept of shadow actuation to actuate the limb indirectly. Other devices actuate the knee joint indirectly by forcing the shank via a linear actuator [3,4], and a push-rod [5] to mimic the biomechanics of the knee joint. Other devices, such as those actuated using pneumatic artificial muscles (PAMS) [6,7], hydraulic actuators [8,9], or motors [10,11,12,13], actuate combinations of ankle–knee–hip (AKH) joints only in the sagittal plane. 

Cable-driven rehabilitation devices (CDRD) are known to facilitate remote actuation while respecting the biomechanics of the joints. These devices are typically lighter weight and exert negligible inertia and inertial vibration on the impaired limb. Furthermore, unlike direct actuation-based design, CDRDs do not require exact alignment with the joints; thus, they reduce donning on/off time with enhanced safety. In the past few decades, several pioneer CDRD devices were proposed. These include C-ALEX [14,15], ROPES [16], MCLR [17], and the two cable-driven exoskeletons suggested by Kirby et al. [18] for the lower limb. Despite many advantages, the majority of these devices could only provide rehabilitation in the sagittal plane. ROPES [3] is equipped with cable routing in the frontal plane, although the model was analyzed only in the sagittal plane. In our previous work, we proposed C-LREX (Cable-Driven Lower Limb Rehabilitation EXoskeleton) [19,20], providing conceptual models with cable routing only in the sagittal plane.

In the majority of lower limb exoskeletons (either for direct or indirect actuation-based devices), frontal plane motion (hip adduction) was either ignored or allowed passively. The limitation of restricting the motion to the sagittal plane is usually reasonable since a large amount of the motion impairment lies in the sagittal plane. This assumption, however, is not always justifiable, particularly in neurological patients, such as post-stroke patients, where hip circumduction and hip hiking in the frontal plane are quite common. Simplified models of the lower limb (such as a planar-based model) and rigid link-based designs of exoskeletons result in a mismatch between the exoskeleton and human movement, thus affecting the overall rehabilitation process [21]. Furthermore, training only for sagittal plane balance compromises frontal plane balance [22]; thus, combined bi-planar training should be preferred in rehabilitation. Previous work on multi-planar cable-driven devices can be mainly found for upper limb rehabilitation, including CAREX [23], CAREX-7 [24], and the upper arm cable-driven system by Chen et al. [25] and Herbin et al. [26]. 

Clinical tests with Lokomat [27] revealed that early training with a rehabilitation devices is likely to improve the ROM and torque generation capability of joints along with reduced stiffness of impaired limbs. Furthermore, the experimental test with C-ALEX [14] improved the capability of the impaired limb to track the reference ankle trajectory. Motivated by promising clinical results and considering the planar-based and link-driven limitations, this work extends the previous sagittal plane-based models for C-LREX and C-ALEX to accommodate motion in the frontal plane (mainly Hip Adduction/Abduction) and provides a general methodology for analysis. Moreover, the feasibility of employing bi-planar cable routing at the hip joint to track the bi-planar reference healthy trajectory using the developed 3DOF model is investigated here. Section 2 of this paper presents the 3DOF lower-limb dynamic models for C-LREX. Section 3 focuses on the bi-planar routing of hip cables based on optimization approaches and its impact on trajectory tracking. Modified cable routing for bi-planar trajectory tracking is also explored, where the effect of different routing on trajectory tracking is discussed, and a modified four-cable routing is proposed. Section 4 summarizes the model, results, and limitations, followed by conclusions and future work.

## 2. The 3DOF Model

**Figure 1:**
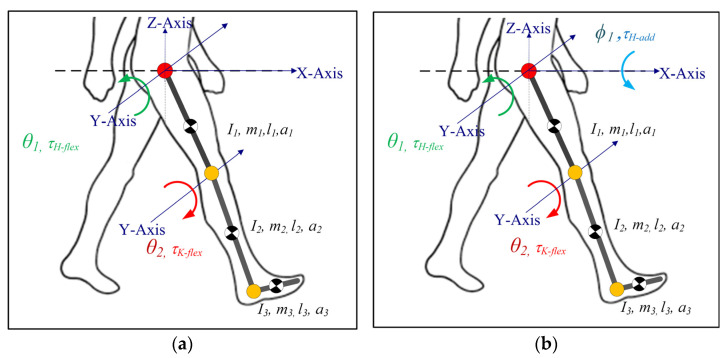
Lower limb model for C-LREX (**a**) 2DOF (**b**) 3DOF (*I*, *m*, *l*, *a* represent the moment of inertia, mass, length of the link, and distance of CG of the link from the joint center. The subscripts *1*, *2*, and *3* refer to link 1 (thigh), link 2 (shank), and link 3 (foot)).

In our previous work [19,20], the 2DOF lower limb model only simulated hip and knee flexion/extension motion (sagittal plane), as shown in Figure 1a. Here, to investigate frontal plane motion and cable routing, while the knee joint was modelled as a 1DOF joint (only Flexion/Extension), the hip joint was modelled as a 2DOF joint (Flexion/Extension and Adduction/Abduction), as depicted in Figure 1b. The internal/external rotation of the hip joint in the transverse plane was neglected in the current model. Furthermore, the current model only simulated the impaired lower limb during the swing phase of gait (the limb is off the ground during the gait cycle), assuming that assistance was only needed during this phase of the motion. The foot was assumed to be perpendicularly fixed to the shank. The rotation of the foot was ignored since the joint moment contribution of the foot during the swing phase was relatively small [28]; however, the inertial properties were kept in the model. 

The dynamic model can be described using Newton Euler’s or Euler Lagrange’s formulation, where $q={\left[\begin{array}{ccc} \phi_{1} & \theta_{1} & \theta_{2} \end{array}\right]}^{T}$ is considered as the generalized coordinate; $\phi_{1}$ represents the hip adduction angle; $\theta_{1}$ and $\theta_{2}$ represent the hip and knee flexion angles, respectively. The generalized equation of the dynamics can then be expressed as:

(1) $$M\left(q\right)\ddot{q}+C\left(q,\dot{q}\right)\dot{q}+G\left(q\right)=\tau$$

where *M(q)* is the inertial matrix ($M\in {\mathbb{R}}^{3\times 3}$); $C\left(q,\dot{q}\right)$ represents the Coriolis component ($C\in {\mathbb{R}}^{3\times 1}$); *G(q)* represents the Gravitational components ($G\in {\mathbb{R}}^{3\times 1}$); and τ represents the torques on the joints ($\tau =[\tau_{H-add}\;\tau_{H-flex}\;\tau_{K-flex}]\in {\mathbb{R}}^{3\times 1}$). The matrices (*M*, *C,* and *G*) are listed in Appendix A.

### 2.1. Generalized Cuff Definition

**Figure 2:**
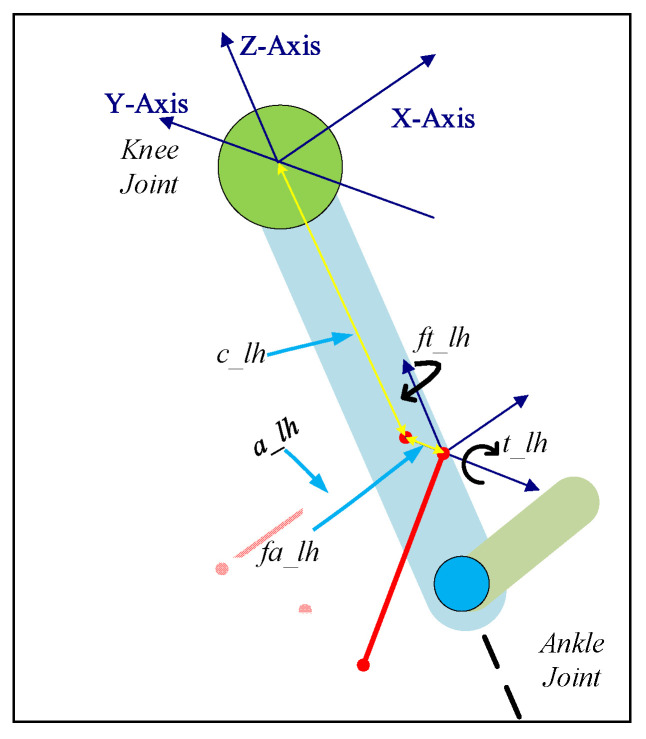
Generalized cuff definition using 5 parameters on Shank.

The cuff was defined using five parameters (refer to Figure 2 and Table 1), including two additional parameters in contrast to the previous 2DOF Model [20]. The sagittal-plane cable routing could be transformed into 3D space by defining additional parameters fa_lh and ft_lh values. With these parameters, any cuff in 3D space can be fully defined.

### 2.2. Force to Joint Torque Mapping

The cable exerts a certain joint moment on the joints depending on the routing and the magnitude of force applied. A vector projection method [19,20] was employed to estimate the Jacobian for each cable. These were then combined to develop the control performance matrix which represents the collective conversion of cable tension to equivalent joint moment.

**Figure 3:**
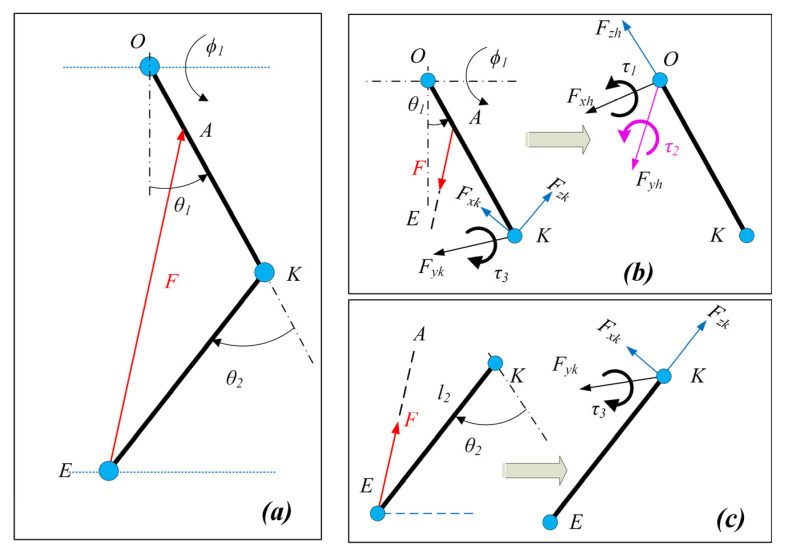
Cable force to joint moment mapping: (**a**) Two-link model with cable tension applied from thigh to shank, (**b**) Equivalent joint moment and component forces acting at the hip joint, and (**c**) Equivalent joint moment and component forces acting at the knee joint.

The unit vectors along the limb sections were estimated (along the *X*, *Y*, and *Z* axis, refer to Figure 2 for directions) using the joint angles of the lower limb and were dependent on the geometric configurations. The cable tension vector was estimated along the cable with applied cable tension and was projected on the unit vectors to estimate the equivalent joint component forces (Figure 3). Furthermore, the equivalent joint moment was obtained by the cross-product of the distance vector and cable tension vector.

Assuming ${\hat{u}}_{SH-x},{\hat{u}}_{SH-y},{\hat{u}}_{SH-z}$ are the unit vectors estimated for the shank rigid segment, then the joint component forces and joint torque were estimated as:

(2) $$\begin{array}{l} \vec{F_{zk}}=(proj_{{\hat{u}}_{SH-z}}\vec{F_{EA}})=(({\hat{u}}_{SH-z}•\vec{F_{EA}}){\hat{u}}_{SH-z}),\vec{F_{yk}}=(proj_{{\hat{u}}_{SH-y}}\vec{F_{EA}})=(({\hat{u}}_{SH-y}•\vec{F_{EA}}){\hat{u}}_{SH-y})\\ \vec{F_{xk}}=(proj_{{\hat{u}}_{SH-x}}\vec{F_{EA}})=(({\hat{u}}_{SH-x}•\vec{F_{EA}}){\hat{u}}_{SH-x}),\left[\begin{array}{ccc} 0 & \vec{\tau_{3}} & 0 \end{array}\right]=(\vec{KE}\times \vec{F_{EA}}) \end{array}$$

where $\vec{F_{EA}}=F_{EA}•{\hat{u}}_{EA}$ is the cable tension vector.

Similarly, the joint force components and joint torque acting at the hip joint can be estimated. Assuming ${\hat{u}}_{TH-x},{\hat{u}}_{TH-y},{\hat{u}}_{TH-z}$ are the unit vectors estimated for the thigh rigid segment, the joint component forces estimated for the shank contributed to the joint component forces as well as the joint moment as:

(3) $$\begin{array}{l} \vec{F_{zh}}=(proj_{{\hat{u}}_{TH-z}}\vec{F_{zk}})+(proj_{{\hat{u}}_{TH-z}}\vec{F_{yk}})+(proj_{{\hat{u}}_{TH-z}}\vec{F_{xk}})+(proj_{{\hat{u}}_{TH-z}}\vec{F_{AE}})\\ \vec{F_{xh}}=(proj_{{\hat{u}}_{TH-x}}\vec{F_{zk}})+(proj_{{\hat{u}}_{TH-x}}\vec{F_{yk}})+(proj_{{\hat{u}}_{TH-x}}\vec{F_{xk}})+(proj_{{\hat{u}}_{TH-x}}\vec{F_{AE}})\\ \vec{F_{yh}}=(proj_{{\hat{u}}_{TH-y}}\vec{F_{zk}})+(proj_{{\hat{u}}_{TH-y}}\vec{F_{yk}})+(proj_{{\hat{u}}_{TH-y}}\vec{F_{xk}})+(proj_{{\hat{u}}_{TH-y}}\vec{F_{AE}})\\ \left[\vec{\tau_{1}}\;\vec{\tau_{2}}\;0]=(\vec{OK}\times \vec{F_{zk}})+(\vec{OK}\times \vec{F_{yk}})+(\vec{OK}\times \vec{F_{xk}})+(\vec{OA}\times \vec{F_{AE}})+[0\;\vec{\tau_{3}}\;0\right] \end{array}$$

The only unknown in Equations (2) and (3) was the magnitude of the cable tension. The unit vectors along limb sections and cables were determined from the geometry. 

Based on Equations (2) and (3), the relation between cable tension and joint moment for a cable is written as:

(4) $$\tau_{cable}=\left[\begin{array}{c} \tau_{1}\\ \tau_{2}\\ \tau_{3} \end{array}\right]=\left[\begin{array}{c} J_{11}\\ J_{21}\\ J_{31} \end{array}\right]F=J^{T}F$$

If the cuff locations are known, the relation between cable tension and joint moment for four cables can be found as:

(5) $$\begin{array}{l} F={\left[\begin{array}{cccc} F_{1} & F_{2} & F_{3} & F_{4} \end{array}\right]}^{T},\tau_{cable}={\left[\begin{array}{ccc} \tau_{1} & \tau_{2} & \tau_{3} \end{array}\right]}^{T}\\ B={\left[\begin{array}{cccc} J_{1} & J_{2} & J_{3} & J_{4} \end{array}\right]}^{T}={\left[\begin{array}{cccc} J_{11} & J_{12} & J_{13} & J_{14}\\ J_{21} & J_{22} & J_{23} & J_{24}\\ J_{31} & J_{32} & J_{33} & J_{34} \end{array}\right]}^{T}\\ \tau_{cable}=B^{T}F\\ B\in {\mathbb{R}}^{4\times 3},J\in {\mathbb{R}}^{3\times 1} \end{array}$$

One of the key problems in cable-driven mechanisms is to ensure the tautness of the cables. The minimum cable tension was limited to 7N so that the cable was always taut, while the maximum cable tension should be limited to ensure that the maximum moment applied to the joint is within a predefined range. The exoskeleton works as an assistive device interfacing with humans; hence, the assistive moment that C-LREX can apply is limited to a moment corresponding to 100N. Depending upon the number of cables, the control performance matrix *B* could be square or rectangular, and it may not be easy to estimate the cable tension based on the required joint moment. Thus, the cable tension distribution problem was formulated as a hybrid optimization problem of error minimization (primary) and control effort minimization (secondary), where QP (quadratic programming) was employed for the solution. The hybrid optimization problem solution guaranteed the existence of a solution in all possible scenarios (particularly when no unique solution exists in the primary case) [29]. Moreover, a PD controller with the same three-layer control architecture [20] as the 2DOF model was employed to track the healthy trajectory as a reference. 

The user’s voluntary contribution (joint moment) in the sagittal plane represents the passive-elastic joint moment [30] (produced by the ligaments, and other tissues around the joint) and was included in the 3DOF model, while no contribution was assumed in the frontal plane. The anthropometric data (shown in Table 2) were adopted from Winter’s [31] (based on body weight and height), where the moment of inertia in the frontal plane was considered the same as that in the sagittal plane. The reference trajectory was based on Fukuchi’s [28] data (overground walking of 3.48 s gait cycle time). 

## 3. Trajectory Tracking with Four-Cable Configuration

**Figure 4:**
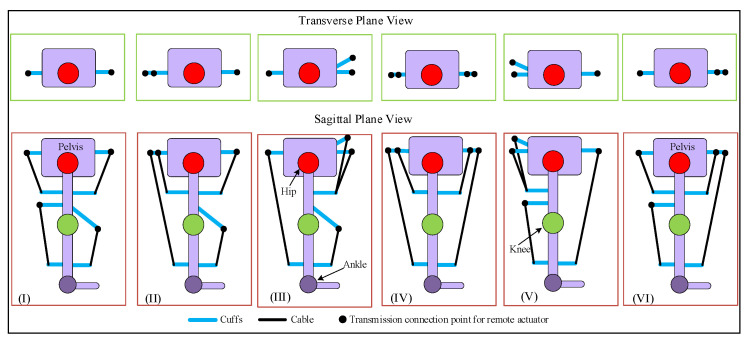
Possible routings for 4-cable driven C-LREX constraining 2 cables for the knee joint (transverse and sagittal planes view).

For an open-chain cable-driven mechanism, the number of cables must be higher than the DOFs being driven [32,33] in order to fully constrain the motion. In our recent work [20], the motion of the 2DOF model-based design of C-LREX was fully constrained by using three cables. In this work, we employed four cables to track the bi-planar trajectory of the lower limb. Although four cables should be sufficient to successfully constrain the motion of a 3DOF model of the lower limb, the configuration with which the cables were routed around the lower limb was also relevant. The possible routing configurations for four cables around the lower limb for C-LREX are shown in Figure 4. These configurations constrained two cables for the knee joint in each possible routing to generate flexion/extension moment at the knee. The antagonistic routing of cables around the knee joint guaranteed tracking of the knee joint trajectory. Thus, the focus would be on tracking the bi-planar trajectory at the hip level. The cables were driven by motors located at a remote location. 

Cases (IV), (V), and (VI) were excluded from the simulation analysis since the anterior cable (the long cable spanning both the hip and knee joint) is difficult to route practically, especially at the higher knee and hip flexion angles. Furthermore, such routing requires larger hinge requirements on the limbs to ensure a safe distance between the cable and the knee joint throughout the range of motion. Case (I) routing employed two cables to track the 2DOF motion (equal number of cables and DOF being tracked) and was thus excluded from further analysis. Case (II) and (III) were practically feasible to route and were studied with the 3DOF model to explore the feasibility of tracking the bi-planar healthy trajectory.

**Figure 5:**
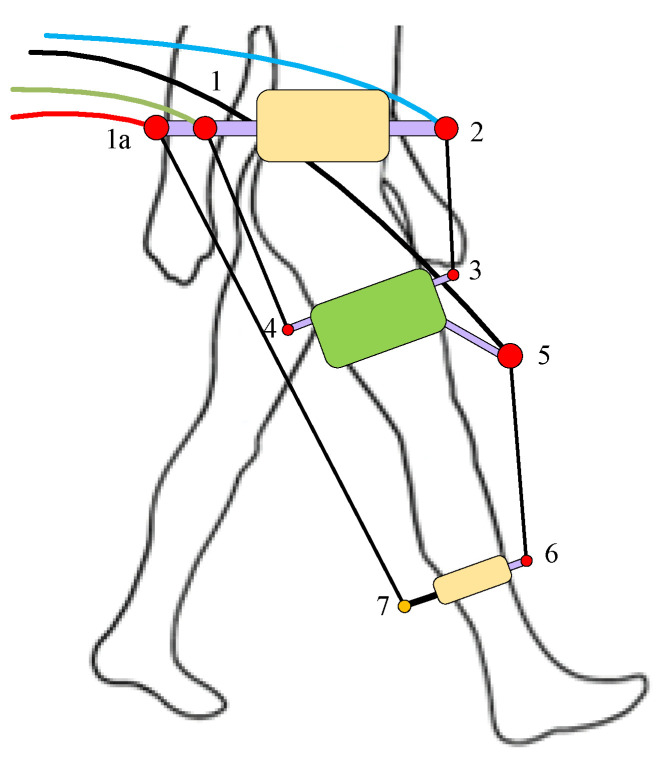
Conceptual configuration of C-LREX with 4 cables case (II) routing. Numbers denote hinges for the cable attachment.

Case (II) was capable of generating both the positive and negative joint moments at each joint in the sagittal plane only (conceptual configuration shown in Figure 5). The cable hinge parameters are listed in Table 3. The parameters *fa_lh* and *ft_lh* were zero due to sagittal plane routing. 

### 3.1. Trajectory Tracking with C-LREX Case (II) 

**Figure 6:**
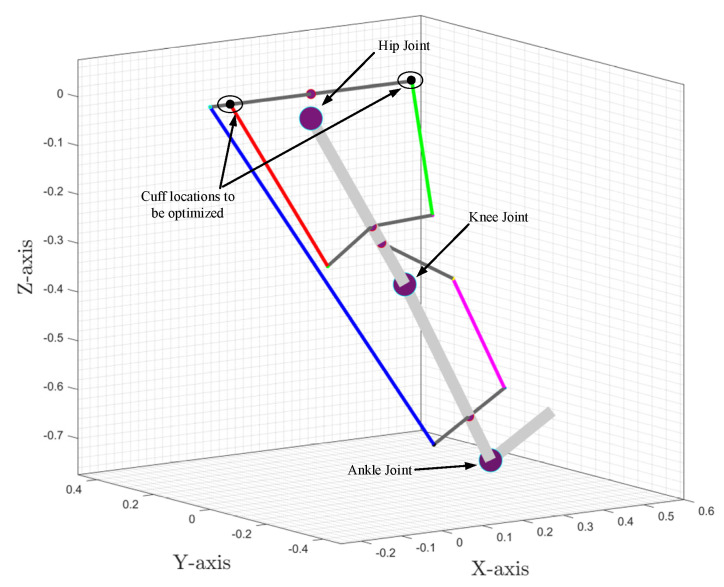
The exoskeleton and 3D space for optimal cuff location.

In the 3DOF model for the hip joint, the moment was to be generated for both sagittal and frontal plane motions, and hence the cable routing in case (II) was modified. Moreover, only the locations of the hip-level cuff hinges were modified (hinges 1 and 2 in Figure 5) to satisfy the bi-planar joint moment requirement at the hip level (as shown in Figure 6). The long cable in the posterior (joining hinges 1a and 7) was kept in the sagittal plane so that only sagittal plane torque was generated at the knee joint. The remaining cuffs were also maintained in the sagittal plane. 

**Figure 7:**
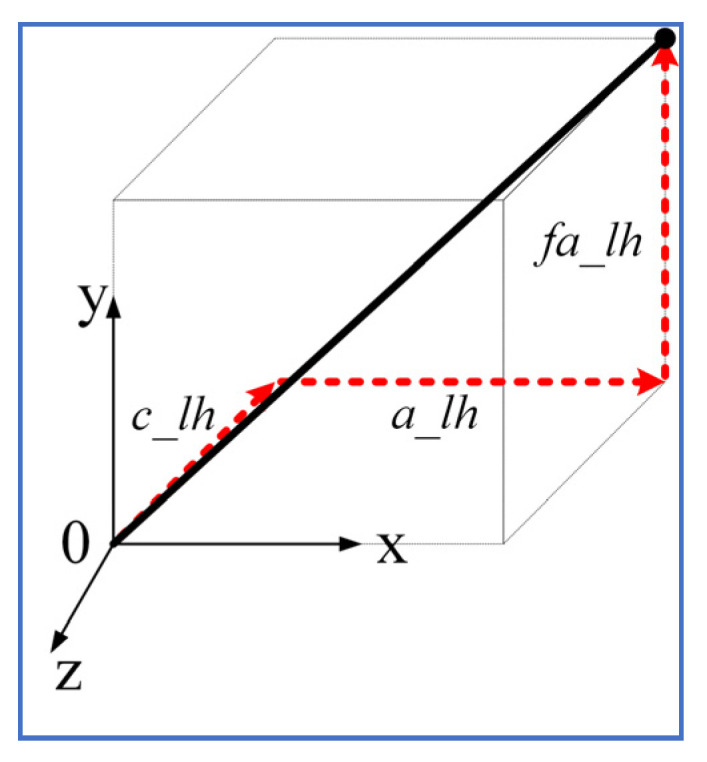
Cuff end location estimation along axes.

To find the suitable location for the upper cuffs (hinges 1 and 2) that can meet the model demand for both sagittal and frontal plane moments’ demand, we formulated an optimization problem to identify the optimal location of the upper cuffs (hinges 1 and 2) for the entire gait cycle. Since the model needed to optimize the upper cuff location only, the coordinates of the upper cuffs were included as variables. A cuff end location is typically dependent on five variables which can be arranged in multiple ways. To reduce the optimization variables for each cuff from five to three, the cuff end location (thick black line in Figure 7) was considered along the axes (red lines in Figure 7), i.e., the angular orientation of the cuff about the axes was fixed.

Assuming ${\left[\begin{array}{ccc} x_{1} & y_{1} & z_{1} \end{array}\right]}^{T}$ and ${\left[\begin{array}{ccc} x_{2} & y_{2} & z_{2} \end{array}\right]}^{T}$ are the positions of the upper cuff on the posterior and anterior sides of the hip, the sum of the *l*_2_ norm of the errors was adopted to obtain a scalar objective function as:

(6) $$\begin{array}{l} \min\left(\left\|e_{hipA}\right\|+\left\|e_{hipF}\right\|+\left\|e_{kneeF}\right\|\right)\\ s.t.\left\{\begin{array}{l} x_{\min}\leq x\leq x_{\max}\\ y_{\min}\leq y\leq y_{\max}\\ z_{\min}\leq z\leq z_{\max} \end{array}\right. \end{array}$$

where $e_{hipA},e_{hipF},e_{kneeF}$ represent errors in joint angle tracking in hip adduction, hip flexion, and knee flexion, respectively. 

The range of values for the variables in the optimization problem is listed in Table 4. MATLAB-based *fmincon* function was employed to solve the above optimization problem.

The above optimization problem converged on the following optimal solution for the hip cuff locations:

$${\left[x_{1}\;y_{1}\;z_{1}\;x_{2}\;y_{2}\;z_{2}\right]}^{T}=X_{opt}={\left[-0.15\;-0.10\;-0.1227\;-0.30\;-0.0557\;-0.2\right]}^{T}$$

**Figure 8:**
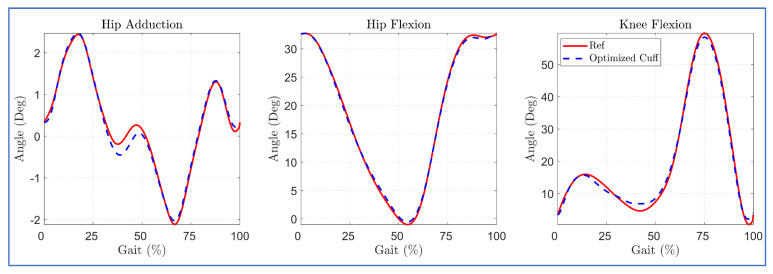
Trajectory tracking with case (II) optimized cuff configuration.

**Figure 9:**
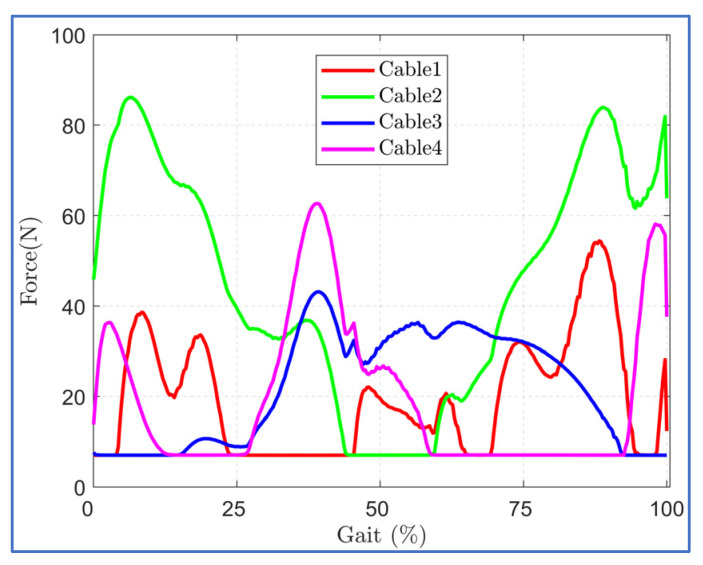
Cable Tension requirements in case (II) optimized cuff configuration.

The trajectory tracking with optimized cuff location is shown in Figure 8. The optimal cuff location allowed close tracking of the reference bi-planar trajectory with minor deviation in hip adduction trajectory during the mid of the gait cycle. This is possibly due to the routing of the hip cables, each in the anterior and posterior zone, since the cable tension requirement during trajectory tracking was well within the imposed limit of 7–100 N during the middle of the gait cycle (Figure 9).

### 3.2. Trajectory Tracking with C-LREX Based on Modified Cable Routing (Case (III))

**Figure 10:**
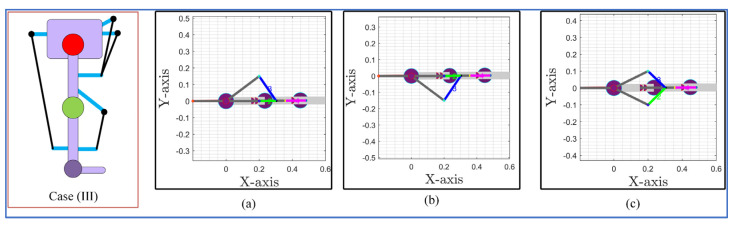
Case (III) and its sagittal and transverse plane view for different routings: (**a**) the 1 cable bi-planar routing (medial side), (**b**) the 1 cable bi-planar routing (lateral side), and (**c**) the 2 cables bi-planar (medial and lateral) routing.

Case (III) routing (in Figure 4) included two cables on the anterior side and one cable on the posterior side at the hip joint level. The anterior cables could be routed in various possible ways. Firstly, for the hip side anterior cables, one of the cables was kept in the sagittal plane while the other was routed in a bi-planar manner (both the sagittal and frontal). The bi-planar cable could be routed on either side of the sagittal axis (medial side or lateral side of the hip joint), as shown in Figure 10a,b. Later, for the hip-side anterior cables, both cables were routed in a bi-planar manner (mirror image of each other with respect to the sagittal plane as shown in Figure 10c). The posterior hip cable and anterior knee cable were kept the same for all configurations in Figure 10. 

**Figure 11:**
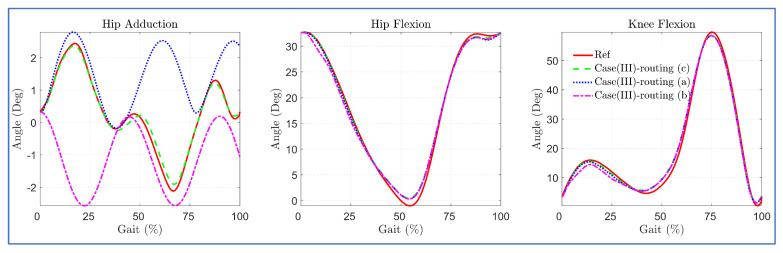
Trajectory tracking with various ways of cable routing of the case (III) of 4-cable configuration.

The simulation with the 3DOF model for case (III) configurations indicated that bi-planar routing of hip cables on the anterior side successfully tracked the reference bi-planar trajectory, while the single bi-planar cable routing failed to track the trajectory, as shown in Figure 11. The routing of two bi-planar cables at the hip joint enabled C-LREX to track frontal as well as sagittal plane trajectories due to the capability to generate both positive and negative moments in the frontal plane.

### 3.3. Influence of Routings on Joint Force Component

**Figure 12:**
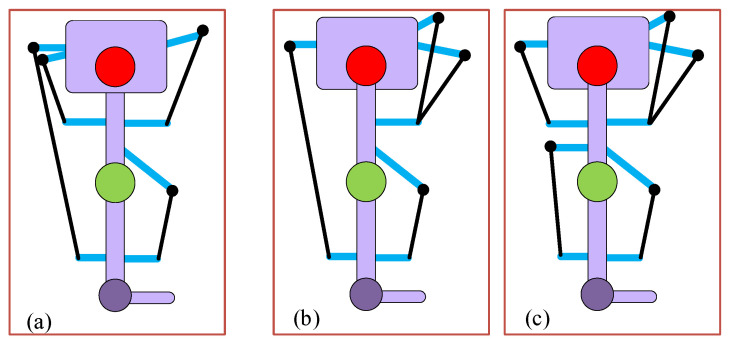
Cable routings: (**a**) The 4-cable case (II) routing, (**b**) The 4-cable case (III)-c routing, and (**c**) The 5-cable routing.

In our previous work [20], it was observed that implementing long cables (combined for the hip and knee joints) exerts higher joint component forces at the physiological joints. To analyze the impact of the four cables in case (II) optimized cuff and case (III)-(c) routing at the hip and knee joint, we proposed a five-cable routing-based design of C-LREX by separating the posterior side cable of the case (III)-(c) routing into two cables (for hip and knee joint separately), as shown in Figure 12. 

**Figure 13:**
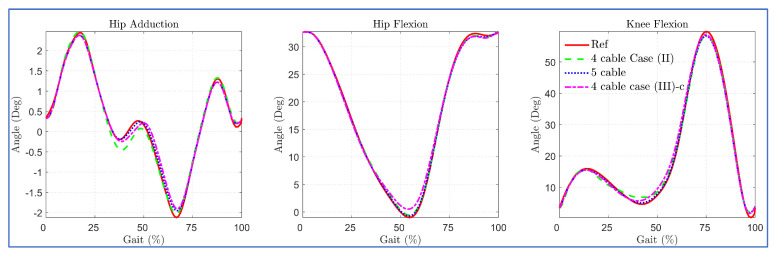
Tracking errors in 4 cables versus 5 cables routing.

The simulation results with the 3DOF model for different routings revealed that the five-cable routing tracked the bi-planar reference trajectory with the least deviation, as shown in Figure 13. 

**Figure 14:**
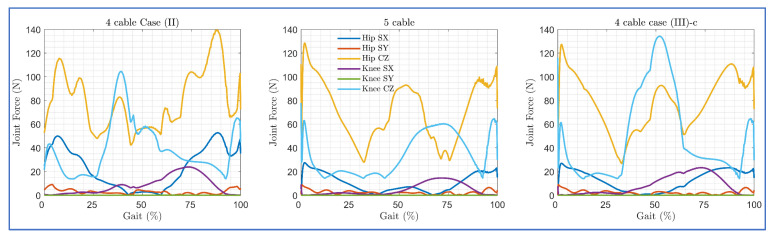
Joint force components induced by the applied cable tensions in each routing (SX and SY refer to shear forces along *X* and *Y* axis, CZ refers to compressive force along *Z* axis).

The maximum applicable cable tension was constrained to 100N during the simulation for both routings. The induced joint force components in each routing are shown in Figure 14. Despite these routings resulting in similar trajectory tracking, the magnitude of the induced force components was different. For the initial step of the gait cycle, the angular velocity was assumed to be zero, which resulted in higher cable tension demands and induced higher force components in each routing case. 

Since the joint force components are the external forces that the user experiences while using C-LREX, higher values may result in discomfort to the user. For the knee joint, except for the initial gait cycle zone, the four-cable case (III)-c and case (II) routings exerted approximately 120% and 70% higher compressive force (Knee CZ), respectively, compared to five-cable routing. Furthermore, case (II) routing exerted higher (8% approximately) peak hip compressive force (Hip CZ) compared to five-cable routing. Case (II) and case (III)-c both exerted approximately 70% higher shear force (Knee SX) as compared to five-cable routing. 

## 4. Discussion

The majority of current lower-limb rehabilitation cable-driven exoskeletons operate only in the sagittal plane. Hence, they are unable to address movement dysfunction in the other planes of motion. These often occur as a result of various neurological pathologies, including stroke and Parkinson’s Disease. In this work, we developed a 3DOF bi-planar cable-driven exoskeleton model by incorporating hip adduction/abduction motion in the frontal plane. The model used a three-link pendulum model for the lower limbs, in which the inertial and dimensional parameters of the limbs were estimated using Winter’s model based on the user’s anthropometric properties. Although the alterations in these parameters influence the anthropometric data, the model dynamics remained unchanged. The physiological gait cycle was adopted from Fukuchi’s work dataset at a slower speed (longer walking time) since walking speed is typically reduced in stroke survivors. An increase in the walking speed would increase the power demand at the joints and vice versa. 

The study aimed to assess the possibility of tracking a bi-planar trajectory with four cable routing configurations without the inclusion of additional cables. Six different potential routings were generated by subjecting two cables for the knee joint in an antagonistic configuration. Due to their impracticality, three of these routing configurations were excluded from the analysis as they intersected the knee joint. Furthermore, case (I) was also excluded from the analysis as it had the same number of cables as the controlled DOF at the hip joint. Case (II) routing of four cables was only capable of successfully generating flexion/extension joint moments in the sagittal plane. To generate a bi-planar moment at the hip joint, we modified case (II) routing for the hip upper-cuff location via an optimization problem which estimated the optimal location of the anterior and posterior hip cuffs for the hip cable for the whole gait cycle. The optimized cuff location-based case (II) routing tracked the reference trajectory closely with a slight deviation in the frontal plane trajectory. Despite the bi-planar routing of two hip cables, the deviation is due to routing each cable on the anterior and posterior sides. 

We further simulated the modified routing of four cables (case (III)) with the 3DOF model. The routing successfully tracked the bi-planar trajectory when two anterior cables on the hip side were routed in a bi-planar manner. However, when one of the hip side anterior cables was kept planar in the sagittal plane, the model failed to track the bi-planar trajectory. The hip posterior side cable was routed so that it spanned both the hip and knee joints. The motivation for limiting the number of cables to four was to track the bi-planar trajectory with the smallest possible number of cables (towards a lightweight, compact exoskeleton). Moreover, we also investigated the effect of an additional cable (five cables) on tracking and joint force components. We found that adding an extra cable to the configuration induced smaller joint force components while tracking bi-planar trajectory but required additional motor power.

## 5. Conclusions

Due to their simple design, lightweight, remote actuation, and easy–safe user interface, cable-driven rehabilitation devices have multiple advantages over traditional link-based devices. However, up to date, CDRDs have failed to address frontal plane motion dysfunction associated with various neurological diseases such as stroke, as they are limited to the sagittal plane only. In this work, we developed a 3DOF lower limb model based on our previously published planar C-LREX 2DOF model by adding frontal plane motion to the hip joint (abduction/adduction). We implemented the 3DOF model to track bi-planar trajectory employing various routings of four-cable configurations. 

We modified the case (II) routing for the hip upper-cuff location via optimization problems to estimate the optimal cuff locations considering the entire gait cycle. The optimal cuff location tracked the bi-planar trajectory with a slight deviation in the frontal plane trajectory. A modified four-cable routing (case (III)-c) successfully tracked the bi-planar trajectory when the two cables of the anterior side were routed in a bi-planar manner. The modified four-cable routing and optimized case (II) routing, however, exerted higher joint force components at the hip and knee joints with similar joint angle tracking as compared with five-cable routing. 

The model currently ignored the active contribution of the user’s limb which can be added to the passive-elastic joint moment based on impaired gait kinematics/kinetics information. The current study was limited to four cable-based configurations. Further investigation of possible solutions to track bi-planar trajectory exploring the number of cables, routings, and configurations will be conducted in future work. 

## Figures and Tables

**Table 1:** Generalized Cuff Parameters Definition.

Name	Details
*c_lh*	Distance of the cuff from the joint center along the *Z*-axis
*a_lh*	Distance of the cuff end from the limb central axis along the *X*-axis
*fa_lh*	Distance of the cuff base from the limb central axis along the *Y*-axis
*t_lh*	Rotation of the cuff about the *Y*-axis
*ft_lh*	Rotation of the cuff about the *Z*-axis

**Table 2:** Anthropometric data included in the study.

Parameter	Value	Unit	Parameter	Value	Unit
*m*_1_	7.58	kg	*I*_1_	0.1527	kg.m^2^
*m*_2_	3.52	kg	*I*_2_	0.0606	kg.m^2^
*m*_3_	1.1	kg	*I*_3_	0.0075	kg.m^2^
*l*_1_	0.44	m	*a*_1_	0.1902	m
*l*_2_	0.43	m	*a*_2_	0.1879	m
*l*_3_	0.17	m	*a*_3_	0.0870	m

**Table 3:** Cable hinges parameters.

Hinge Number	Cuff Parameters				
	Length (m)			Angle (Deg)	
	*c_lh*	*a_lh*	*fa_lh*	*t_lh*	*ft_lh*
1	0.0500	0.2000	0	180	0
1a	0.0500	0.2500	0	180	0
2	0.0500	0.2000	0	0	0
3	−0.2820	0.1500	0	30	0
4	−0.2820	0.1300	0	180	0
5	−0.3254	0.2000	0	60	0
6	−0.3295	0.1000	0	0	0
7	−0.3295	0.1000	0	180	0

**Table 4:** Optimization variable ranges.

Variable (Unit)	Min Value	Max Value
*x*_1_ (m)	−0.3	−0.15
*y*_1_ (m)	−0.1	0.1
*z*_1_ (m)	0	0.2
*x*_2_ (m)	0.15	0.3
*y*_2_ (m)	−0.1	0.1
*z*_2_ (m)	0	0.2

## Data Availability

Data available on request.

## Appendix A

Inertial matrix (M)

(A1) $$\begin{array}{l} M\left(q\right)=\left[\begin{array}{ccc} M_{11} & 0 & 0\\ 0 & M_{22} & M_{23}\\ 0 & M_{32} & M_{33} \end{array}\right]\\ M_{11}=\left(m_{1}{a_{1}}^{2}\cos{\left(\theta_{1}\right)}^{2}+m_{2}{\left(l_{1}\cos\left(\theta_{1}\right)+a_{2}\cos\left(\theta_{1}-\theta_{2}\right)\right)}^{2}+m_{3}{\left(l_{1}\cos\left(\theta_{1}\right)+l_{2}\cos\left(\theta_{1}-\theta_{2}\right)-a_{3}\sin\left(\theta_{1}-\theta_{2}\right)\right)}^{2}+I_{1,x}+I_{2,x}+I_{3,x}\right)\\ M_{22}=\left(\begin{array}{l} \left(\frac{\cos\left(2\phi_{1}\right)}{2}+\frac{1}{2}\right)\left(I_{1,y}+I_{2,y}+I_{3,y}\right)+\left(\frac{1}{2}-\frac{\cos\left(2\phi_{1}\right)}{2}\right)\left(I_{1,z}+I_{2,z}+I_{3,z}\right)+m_{3}\left({a_{3}}^{2}+2\sin\left(\theta_{2}\right)a_{3}l_{1}+{l_{1}}^{2}+2\cos\left(\theta_{2}\right)l_{1}l_{2}+{l_{2}}^{2}\right)+\\ m_{1}{a_{1}}^{2}+m_{2}\left({a_{2}}^{2}+2\cos\left(\theta_{2}\right)a_{2}l_{1}+{l_{1}}^{2}\right) \end{array}\right)\\ M_{32}=\left(\left(\frac{\cos\left(2\phi_{1}\right)}{2}-\frac{1}{2}\right)\left(I_{2,z}+I_{3,z}\right)-\left(\frac{\cos\left(2\phi_{1}\right)}{2}+\frac{1}{2}\right)\left(I_{2,y}+I_{3,y}\right)-m_{2}\left({a_{2}}^{2}+l_{1}\cos\left(\theta_{2}\right)a_{2}\right)-m_{3}\left(\begin{array}{l} {a_{3}}^{2}+l_{1}\sin\left(\theta_{2}\right)a_{3}+\\ {l_{2}}^{2}+l_{1}\cos\left(\theta_{2}\right)l_{2} \end{array}\right)\right)\\ M_{23}=M_{32}\\ M_{33}=\left(\left({a_{3}}^{2}+{l_{2}}^{2}\right)m_{3}+m_{2}{a_{2}}^{2}+\left(\frac{\cos\left(2\phi_{1}\right)}{2}+\frac{1}{2}\right)\left(I_{2,y}+I_{3,y}\right)+\left(\frac{1}{2}-\frac{\cos\left(2\phi_{1}\right)}{2}\right)\left(I_{2,z}+I_{3,z}\right)\right) \end{array}$$

Coriolis matrix (C)

(A2) $$\begin{array}{l} C\left(q,\dot{q}\right)\dot{q}={\left[\begin{array}{ccc} C_{1} & C_{2} & C_{3} \end{array}\right]}^{T}\\ C_{1}=-[\begin{array}{l} \left(\frac{I_{1,z}\sin\left(2\phi_{1}\right)}{2}-\frac{I_{1,y}\sin\left(2\phi_{1}\right)}{2}-\frac{I_{2,y}\sin\left(2\phi_{1}\right)}{2}+\frac{I_{2,z}\sin\left(2\phi_{1}\right)}{2}-\frac{I_{3,y}\sin\left(2\phi_{1}\right)}{2}+\frac{I_{3,z}\sin\left(2\phi_{1}\right)}{2}\right){\left(\frac{\partial }{\partial t}\theta_{1}\right)}^{2}\\ +(\begin{array}{l} (\begin{array}{l} {a_{1}}^{2}m_{1}\sin\left(2\theta_{1}\right)+{l_{1}}^{2}m_{2}\sin\left(2\theta_{1}\right)+{l_{1}}^{2}m_{3}\sin\left(2\theta_{1}\right)-{a_{3}}^{2}m_{3}\sin\left(2\theta_{1}-2\theta_{2}\right)\\ +{a_{2}}^{2}m_{2}\sin\left(2\theta_{1}-2\theta_{2}\right)+{l_{2}}^{2}m_{3}\sin\left(2\theta_{1}-2\theta_{2}\right)+2l_{1}l_{2}m_{3}\sin\left(2\theta_{1}-\theta_{2}\right)+2a_{3}l_{1}m_{3}\cos\left(2\theta_{1}-\theta_{2}\right)\\ +2a_{3}l_{2}m_{3}\cos\left(2\theta_{1}-2\theta_{2}\right)+2a_{2}l_{1}m_{2}\sin\left(2\theta_{1}-\theta_{2}\right) \end{array})\left(\frac{\partial }{\partial t}\phi_{1}\right)\\ +\left(I_{2,y}\sin\left(2\phi_{1}\right)-I_{2,z}\sin\left(2\phi_{1}\right)+I_{3,y}\sin\left(2\phi_{1}\right)-I_{3,z}\sin\left(2\phi_{1}\right)\right)\frac{\partial }{\partial t}\theta_{2} \end{array})\frac{\partial }{\partial t}\phi_{1}\\ +\left(\frac{I_{2,z}\sin\left(2\phi_{1}\right)}{2}-\frac{I_{2,y}\sin\left(2\phi_{1}\right)}{2}-\frac{I_{3,y}\sin\left(2\phi_{1}\right)}{2}+\frac{I_{3,z}\sin\left(2\phi_{1}\right)}{2}\right){\left(\frac{\partial }{\partial t}\theta_{2}\right)}^{2}\\ +(\begin{array}{l} {a_{3}}^{2}m_{3}\sin\left(2\theta_{1}-2\theta_{2}\right)-{a_{2}}^{2}m_{2}\sin\left(2\theta_{1}-2\theta_{2}\right)-{l_{2}}^{2}m_{3}\sin\left(2\theta_{1}-2\theta_{2}\right)-l_{1}l_{2}m_{3}\sin\left(2\theta_{1}-\theta_{2}\right)\\ -a_{3}l_{1}m_{3}\cos\left(2\theta_{1}-\theta_{2}\right)-2a_{3}l_{2}m_{3}\cos\left(2\theta_{1}-2\theta_{2}\right)-a_{3}l_{1}m_{3}\cos\left(\theta_{2}\right)\\ +a_{2}l_{1}m_{2}\sin\left(\theta_{2}\right)-a_{2}l_{1}m_{2}\sin\left(2\theta_{1}-\theta_{2}\right)+l_{1}l_{2}m_{3}\sin\left(\theta_{2}\right) \end{array})\frac{\partial }{\partial t}\phi_{1}\frac{\partial }{\partial t}\theta_{2} \end{array}]\\ C_{2}=-[\begin{array}{l} (\begin{array}{l} -m_{2}\left(\frac{\sin\left(2\theta_{1}-2\theta_{2}\right){a_{2}}^{2}}{2}+\sin\left(2\theta_{1}-\theta_{2}\right)a_{2}l_{1}+\frac{\sin\left(2\theta_{1}\right){l_{1}}^{2}}{2}\right)+\\ m_{3}\left(\frac{\sin\left(2\theta_{1}-2\theta_{2}\right){a_{3}}^{2}}{2}-\cos\left(2\theta_{1}-\theta_{2}\right)a_{3}l_{1}-\cos\left(2\theta_{1}-2\theta_{2}\right)a_{3}l_{2}-\frac{\sin\left(2\theta_{1}\right){l_{1}}^{2}}{2}-\sin\left(2\theta_{1}-\theta_{2}\right)l_{1}l_{2}-\frac{\sin\left(2\theta_{1}-2\theta_{2}\right){l_{2}}^{2}}{2}\right)-\frac{{a_{1}}^{2}m_{1}\sin\left(2\theta_{1}\right)}{2} \end{array}){\left(\frac{\partial }{\partial t}\phi_{1}\right)}^{2}\\ +(\begin{array}{l} \left(I_{1,y}\sin\left(2\phi_{1}\right)-I_{1,z}\sin\left(2\phi_{1}\right)+I_{2,y}\sin\left(2\phi_{1}\right)-I_{2,z}\sin\left(2\phi_{1}\right)+I_{3,y}\sin\left(2\phi_{1}\right)-I_{3,z}\sin\left(2\phi_{1}\right)\right)\frac{\partial }{\partial t}\theta_{1}-\\ \left(I_{2,y}\sin\left(2\phi_{1}\right)-I_{2,z}\sin\left(2\phi_{1}\right)+I_{3,y}\sin\left(2\phi_{1}\right)-I_{3,z}\sin\left(2\phi_{1}\right)\right)\frac{\partial }{\partial t}\theta_{2} \end{array})\frac{\partial }{\partial t}\phi_{1}\\ +\left(m_{3}\left(2l_{1}l_{2}\sin\left(\theta_{2}\right)-2a_{3}l_{1}\cos\left(\theta_{2}\right)\right)+2a_{2}l_{1}m_{2}\sin\left(\theta_{2}\right)\right)\frac{\partial }{\partial t}\theta_{1}\frac{\partial }{\partial t}\theta_{2}\\ +\left(-l_{1}m_{3}\left(l_{2}\sin\left(\theta_{2}\right)-a_{3}\cos\left(\theta_{2}\right)\right)-a_{2}l_{1}m_{2}\sin\left(\theta_{2}\right)\right){\left(\frac{\partial }{\partial t}\theta_{2}\right)}^{2} \end{array}]\\ C_{3}=-[\begin{array}{l} (\begin{array}{l} m_{2}\left(\frac{{a_{2}}^{2}\sin\left(2\theta_{1}-2\theta_{2}\right)}{2}-\frac{a_{2}l_{1}\sin\left(\theta_{2}\right)}{2}+\frac{a_{2}l_{1}\sin\left(2\theta_{1}-\theta_{2}\right)}{2}\right)-\\ m_{3}(\begin{array}{l} \frac{{a_{3}}^{2}\sin\left(2\theta_{1}-2\theta_{2}\right)}{2}-\frac{{l_{2}}^{2}\sin\left(2\theta_{1}-2\theta_{2}\right)}{2}-a_{3}l_{2}\cos\left(2\theta_{1}-2\theta_{2}\right)-\\ \frac{a_{3}l_{1}\cos\left(\theta_{2}\right)}{2}+\frac{l_{1}l_{2}\sin\left(\theta_{2}\right)}{2}-\frac{l_{1}l_{2}\sin\left(2\theta_{1}-\theta_{2}\right)}{2}-\frac{a_{3}l_{1}\cos\left(2\theta_{1}-\theta_{2}\right)}{2} \end{array}) \end{array}){\left(\frac{\partial }{\partial t}\phi_{1}\right)}^{2}+(\begin{array}{l} \left(I_{2,y}\sin\left(2\phi_{1}\right)-I_{2,z}\sin\left(2\phi_{1}\right)+I_{3,y}\sin\left(2\phi_{1}\right)-I_{3,z}\sin\left(2\phi_{1}\right)\right)\frac{\partial }{\partial t}\theta_{2}-\\ \left(I_{2,y}\sin\left(2\phi_{1}\right)-I_{2,z}\sin\left(2\phi_{1}\right)+I_{3,y}\sin\left(2\phi_{1}\right)-I_{3,z}\sin\left(2\phi_{1}\right)\right)\frac{\partial }{\partial t}\theta_{1} \end{array})\frac{\partial }{\partial t}\phi_{1}\\ +\left(-m_{3}\left(-a_{3}l_{1}\cos\left(\theta_{2}\right)+l_{1}l_{2}\sin\left(\theta_{2}\right)\right)-a_{2}l_{1}m_{2}\sin\left(\theta_{2}\right)\right){\left(\frac{\partial }{\partial t}\theta_{1}\right)}^{2} \end{array}] \end{array}$$

Gravitational component matrix (G)

(A3) $$\begin{array}{l} G(q)={\left[G_{1}\;G_{2}\;G_{3}\right]}^{T}\\ G_{1}=g\sin\left(\phi_{1}\right)\left(m_{2}\left(l_{1}\cos\left(\theta_{1}\right)+a_{2}\cos\left(\theta_{1}-\theta_{2}\right)\right)+m_{3}\left(l_{1}\cos\left(\theta_{1}\right)+l_{2}\cos\left(\theta_{1}-\theta_{2}\right)-a_{3}\sin\left(\theta_{1}-\theta_{2}\right)\right)+m_{1}a_{1}\cos\left(\theta_{1}\right)\right)\\ G_{2}=g\cos\left(\phi_{1}\right)\left(m_{2}\left(l_{1}\sin\left(\theta_{1}\right)+a_{2}\sin\left(\theta_{1}-\theta_{2}\right)\right)+m_{3}\left(l_{1}\sin\left(\theta_{1}\right)+a_{3}\cos\left(\theta_{1}-\theta_{2}\right)+l_{2}\sin\left(\theta_{1}-\theta_{2}\right)\right)+m_{1}a_{1}\sin\left(\theta_{1}\right)\right)\\ G_{3}=g\cos\left(\phi_{1}\right)\left(-m_{2}a_{2}\sin\left(\theta_{1}-\theta_{2}\right)-m_{3}\left(l_{2}\sin\left(\theta_{1}-\theta_{2}\right)+a_{3}\cos\left(\theta_{1}-\theta_{2}\right)\right)\right) \end{array}$$
